# Functional hemodynamic imaging markers for the prediction of pathological outcomes in breast cancer patients treated with neoadjuvant chemotherapy

**DOI:** 10.1117/1.JBO.29.6.066001

**Published:** 2024-05-11

**Authors:** Bin Deng, Ailis Muldoon, Jayne Cormier, Nathaniel D. Mercaldo, Elizabeth Niehoff, Natalie Moffett, Mansi A. Saksena, Steven J. Isakoff, Stefan A. Carp

**Affiliations:** a Massachusetts General Hospital, Athinoula A. Martinos Center for Biomedical Imaging, Department of Radiology, Charlestown, Massachusetts, United States; b Harvard Medical School, Boston, Massachusetts, United States; c Massachusetts General Hospital, Breast Imaging Division, Department of Radiology, Boston, Massachusetts, United States; d Massachusetts General Hospital, Institute for Technology Assessment, Boston, Massachusetts, United States; e Massachusetts General Hospital, Cancer Center, Boston, Massachusetts, United States

**Keywords:** diffuse optical tomography, digital breast tomosynthesis, breast cancer, neoadjuvant chemotherapy, therapy monitoring, functional imaging marker

## Abstract

**Significance:**

Achieving pathologic complete response (pCR) after neoadjuvant chemotherapy (NACT) is a significant predictor of increased likelihood of survival in breast cancer patients. Early prediction of pCR is of high clinical value as it could allow personalized adjustment of treatment regimens in non-responding patients for improved outcomes.

**Aim:**

We aim to assess the association between hemoglobin-based functional imaging biomarkers derived from diffuse optical tomography (DOT) and the pathological outcome represented by pCR at different timepoints along the course of NACT.

**Approach:**

Twenty-two breast cancer patients undergoing NACT were enrolled in a multimodal DOT and X-ray digital breast tomosynthesis (DBT) imaging study in which their breasts were imaged at different compression levels. Logistic regressions were used to study the associations between DOT-derived imaging markers evaluated after the first and second cycles of chemotherapy, respectively, with pCR status determined after the conclusion of NACT at the time of surgery. Receiver operating characteristic curve analysis was also used to explore the predictive performance of selected DOT-derived markers.

**Results:**

Normalized tumor HbT under half compression was significantly lower in the pCR group compared to the non-pCR group after two chemotherapy cycles (p=0.042). In addition, the change in normalized tumor ${\mathrm{StO}}_{2}$ upon reducing compression from full to half mammographic force was identified as another potential indicator of pCR at an earlier time point, i.e., after the first chemo cycle (p=0.038). Exploratory predictive assessments showed that AUCs using DOT-derived functional imaging markers as predictors reach as high as 0.75 and 0.71, respectively, after the first and second chemo cycle, compared to AUCs of 0.50 and 0.53 using changes in tumor size measured on DBT and MRI.

**Conclusions:**

These findings suggest that breast DOT could be used to assist response assessment in women undergoing NACT, a critical but unmet clinical need, and potentially enable personalized adjustments of treatment regimens.

## 1 Introduction

Breast cancer is the primary cause of cancer-related mortality in women. In the United States, ∼20,000 patients are diagnosed with stage III locally advanced breast cancers annually.^1^ Neoadjuvant chemotherapy (NACT) has become the standard-of-care treatment and is increasingly being used in patients with earlier-stage operable tumors with the goals of down-staging the disease, improving operability, allowing breast-conserving surgery, and informing subsequent adjuvant therapy treatment decisions.^2^^,^^3^ However, depending on tumor subtypes and treatment regimens, NACT has heterogeneous outcomes.^4^^–^^6^ Although 70% to 80% of patients demonstrate some level of response after treatment, only 8% to 50% achieve a pathologic complete response (pCR),^7^ the only outcome demonstrated to improve disease-free and overall survival.^8^ The development of reliable methods for the early prediction of tumor response to NACT could spare non-responding patients the toxicity associated with ineffective therapy. In addition, in the emerging era of precision medicine, early prediction of NACT outcomes would enable oncologists to offer personalized, response-adjusted treatment regimens. For example, novel targeted therapy agents can be used in patients with human epidermal growth factor receptor 2-positive (HER2+) cancers that are not responding to the standard regimen, and in patients with triple-negative (TN) diseases, lack of response could justify treatment escalation. The long-term survival data from the GeparTrio trial show a significant benefit in switching clinical non-responders from the standard docetaxel, doxorubicin, and cyclophosphamide (TAC) to combined vinorelbine and capecitabine.^9^ Therefore, early predictions of the pathological outcomes of NACT have high clinical value, not only for reducing breast cancer mortality and limiting unnecessary toxicity and expense related to ineffective therapy but also for developing personalized NACT regimens by switching to non-cross-resistant chemotherapy or adding targeted agents.

Imaging plays a fundamental role in the management of breast cancer, especially in the assessment of tumor response to therapy. Changes in tumor size according to the RECIST (Response Evaluation Criteria In Solid Tumors) guidelines are routinely used as a standard measure of treatment response.^10^ Unfortunately, these methods are known to have limited correlations with pathology.^11^ Crucially, the changes in tumor size lagged notably behind changes in underlying tumor physiology, such as vascularization, vascular permeability, cellularity, and metabolism.^12^^–^^14^ In this context, near-infrared optical spectroscopy and tomography (NIRS/DOT) have emerged as functional imaging methods for NACT monitoring.^15^^–^^17^ Taking advantage of the low optical absorption of biological tissue between 650 and 1000 nm, noninvasive NIR light can penetrate several centimeters deep into the tissue, allowing direct quantitative measurements of biochemical tissue components, such as oxy- and deoxy-hemoglobin (HbO and HbR), water, and lipids.^18^^,^^19^ DOT-derived markers, such as total hemoglobin concentration (HbT = HbO + HbR) and tissue oxygenation (${\mathrm{StO}}_{2}=\mathrm{HbO}/\mathrm{HbT}$), can offer insights into tumor oxygen metabolism, perfusion, and proliferation.^20^ Over the years, several NIRS/DOT studies, either using standalone optical imaging devices^21^^–^^31^ or hybrid systems with breast ultrasound,^32^^–^^34^ have reported statistically significant differences between responders versus non-responders as early as after the first cycle of therapy. The consensus from these studies generally indicates early decreases in tumor HbT, a surrogate marker of blood volume and hence angiogenesis, correlate with favorable treatment outcomes, more so in tumors with higher tissue oxygenation levels. Though promising, these prior studies have yet to result in clinical translation of NIRS/DOT for the early prediction of NACT. Thus, there is a need to further improve the sensitivity and specificity in predicting the pathological outcome, particularly pCR, using optical imaging markers. Further, there is a need to develop imaging methods that can assess such markers in a way that can be easily integrated in the clinical workflow, such as by combining optical imaging with mammography, the most accessible and widely used clinical breast imaging method.

In the meantime, our group^35^^–^^39^ and others^40^^–^^44^ have explored the hemodynamic response of breast tissue to fractional mammographic compression as a novel contrast mechanism for optical breast cancer imaging. In a small study conducted with a standalone fast dynamic DOT imager, we were able to show normalization in breast tumor hemodynamics under compression in responders to NACT.^45^ Motivated by these results, we have developed a new generation of our multimodal X-ray mammography and DOT instrumentation,^46^ which was used previously for differential diagnosis of suspicious breast lesions,^47^ to dynamically image breast tissue at different compression levels.

In this work, we study tumor contrast at various time points along the course of the treatment, when breasts are under steady-state mammography-like compression and also subject to stepwise changes in compression force, to evaluate the ability of hemoglobin-based DOT metrics in distinguishing partial and complete pathologic response at the end of the NACT regimen in patients with locally advanced breast cancer.

## 2 Methods

### 2.1 Multimodal Imaging System

Our second-generation tomographic optical breast imaging (TOBI2) system has been described in detail previously.^46^ Briefly, the optical imager uses a hybrid design. The continuous-wave (CW) NIRS sub-system consists of 96 sources (3 sets of 16 690 nm and 16 830 nm sources, frequency-multiplexed between 6.4 and 12.6 kHz illuminated simultaneously at a time for 1 s, resulting in a frame rate of 1/3 Hz) and 32 detectors, providing high spatial and temporal data density. The radio-frequency (RF) NIRS sub-system,^48^ modulated at 67.5 MHz for 690 nm and 75 MHz for 830 nm, consists of 24 time-multiplexed dual-wavelength source locations using a galvo mirror to switch light into one source fiber a time and 20 detectors, providing absolute optical property sampling at the expense of a lower data acquisition rate of 0.1 Hz per 24 source cycle. Both sub-systems use 690 and 830 nm light sources. A schematic drawing is shown in Fig. S1 in the Supplementary Material.

**Fig. 1:**
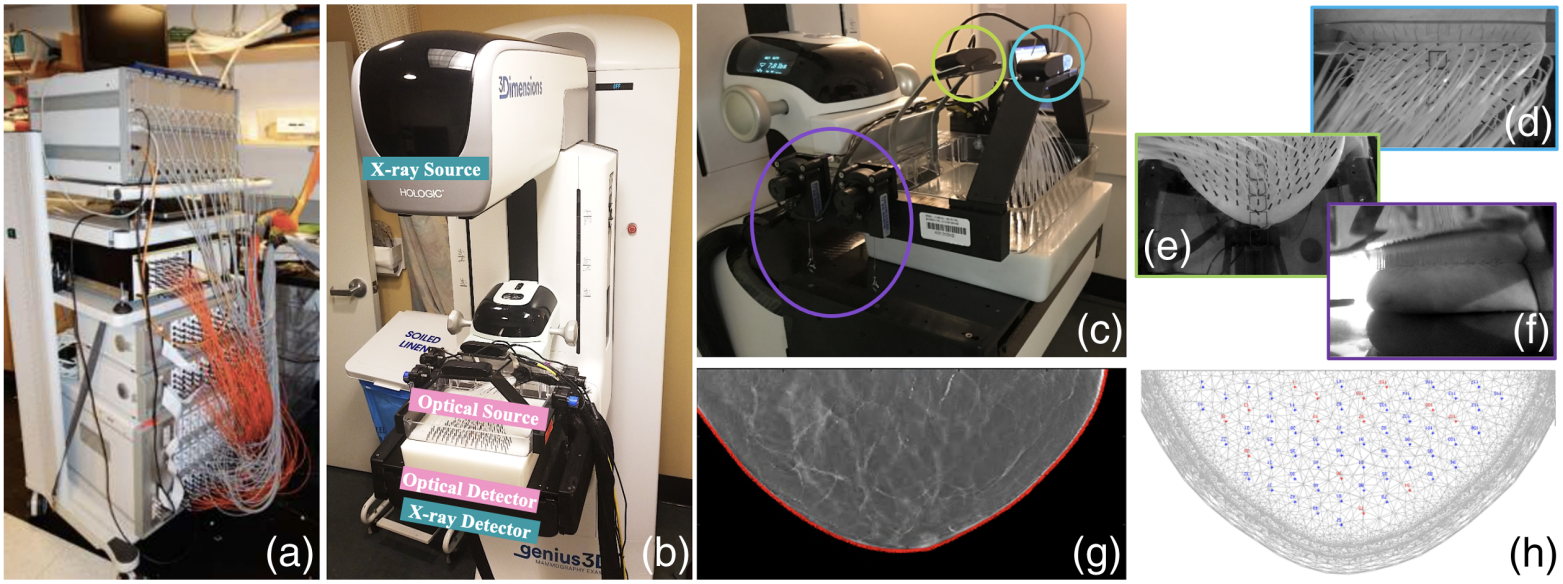
(a), (b) Multimodal DOT/DBT imaging system, (c) including auxiliary devices: webcams (circled in light green and cyan) and linear encoders (purple circle). (d)–(f) Webcams capture breast contours in three views to (h) allow meshing and (g) contour-based image registration with separately acquired DBT.

Optical fibers are mounted in a customized breast compression paddle (source plate) and a low-profile box fitted over the X-ray detector cover (detector plate), respectively. Figure 1 shows these source and detector plates fitted to a 24 cm by 18 cm compression paddle on a Hologic Selenia Dimensions digital breast tomosynthesis (DBT) device, allowing optical data acquisition within an ∼18  cm by 10 cm half-elliptical area under mammographic compression. Three web cameras are mounted on the source plate to capture the breast shape from different angles (two are visible in Fig. 1(c), light green and cyan circles, respectively), later used to create accurate optical image reconstruction meshes. Linear encoders are mounted at four locations on the source plate, two on each side (purple circle in Fig. 1(c)), and attached to hooks on the detector plate during measurement to record accurate source-detector separations and degrees of tilt. Detailed drawings of the arrangement of DOT source and detector optodes and auxiliary devices, i.e., three web cameras and four linear encoders, are shown in Fig. S2 in the Supplementary Material.

### 2.2 Study Design and Patient Eligibility

This study is registered on ClinicalTrials.gov under the ID number NCT03822312. Eligible patients included women 18 years of age or older. Patients were required to have a biopsy-confirmed diagnosis of invasive breast cancer of TN or HER2+ type and be scheduled to receive NACT at Massachusetts General Hospital (MGH) before any excisional surgery. There were no eligibility restrictions based on the chemotherapy agents used in the treatment regimen. In addition, at the time of enrollment, patients must have had measurable disease, defined as at least one lesion that could be accurately measured as 1 cm or greater in the longest dimension with breast MRI, mammography, or ultrasound.

Patients were required to receive multimodal DOT/DBT scans at two time points: before the start of chemotherapy (baseline) and after the second cycle of chemotherapy was complete but right before the beginning of the third cycle (pre-cycle 3). Some patients participated in an additional optional time point, after completing one cycle of chemotherapy but just before the beginning of the second cycle (pre-cycle 2). There was also an option to receive an additional research MRI using clinical breast MRI protocol at pre-cycle 3. Patients were recruited from the MGH Cancer Center. All patient consents were obtained in accordance with the policies and guidelines of the Dana-Farber Harvard Cancer Center Institutional Review Board (IRB), which centralizes regulatory oversight for cancer-related research studies across the affiliated hospitals of Harvard Medical School, including MGH.

### 2.3 Imaging Procedures

**Fig. 2:**
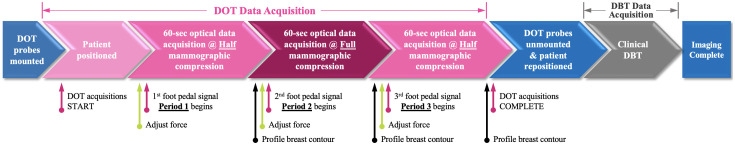
Multimodal DOT/DBT imaging protocol with three stepwise compression force changes.

During the study imaging session, with the optical probes attached to the DBT machine and the patient’s breast compressed in the cranial-caudal (CC) view, three 1-min periods of optical data were acquired at 3-s frame rate with the target force set to approximately half-, full-, then back to half-mammographic compression for each period, respectively, as shown in Fig. 2. The stepwise compression imaging procedure was designed based on our experience from prior studies,^37^^,^^45^ where fractional mammographic compression was shown to generate useful dynamic contrast to differentiate malignant lesions from normal tissue. We included the half-compression period at the beginning in period 1 to match our previous work. Then, we included the standard mammographic force (i.e., full compression), which would offer the easiest path to translation given the familiarity of mammography technicians with full compression. Finally, previous studies have shown that interesting contrast is seen as breast compression decreases due to tissue relaxation. Thus, we included another partial decompression in period 3 to further explore potential contrast mechanisms.

At the end of each optical data acquisition period, photographs were taken of the top and side contours of the breast using the webcams mounted on the source plate. After completing all three optical data acquisition periods, the patient was released from compression, the optical probe plates were removed from the DBT device, and the patient was repositioned for a separate standard DBT imaging at full mammographic compression, also in CC view. The mammography technologist (JC), who conducted both the optical data acquisition and the clinical DBT imaging, used her professional judgment with the help of fiducial markers to consistently position the breast between imaging modalities. However, some shifts and non-elastic deformations were expected and thus compensated for as described below. The breast lesion was later marked on the DBT image stack by a breast radiologist associated with the study (MAS).

Optical image reconstructions were done using a fine tetrahedral mesh to solve the optical forward problem and a coarser tetrahedral mesh to solve the inversion. To generate these meshes, breast contours during DOT acquisition were first extracted from the webcam images, then registered to those extracted from the DBT image stack using a contour-based image registration method described previously,^49^ and finally converted to a set of 3D volumetric meshes by applying a MATLAB-based meshing toolbox, “*iso2mesh.*”^50^ In addition, linear encoder readings were used to correct the tilt of the source plate, which typically resulted in the nipple end being slightly thinner than the opposite end toward the chest wall. To avoid boundary effect distortions in the imaging area, the meshes were extended 2 cm into the chest wall region.

To ensure the accurate quantification of breast tissue optical properties, a raw signal versus source-detector separation plot was made, and only measurements clearly above the electronic noise floor were included in image reconstruction. The maximum effective source-detector separation limit varied between 8 and 12 cm, depending on each patient’s breast optical properties, which are largely driven by breast density. Measurement of a homogeneous phantom of known optical properties was then used to calibrate the raw optical measurements before being fitted for bulk optical properties. An in-house software, *Redbird*, initially developed by Fang et al.,^51^^,^^52^ was used to perform five iterations of a nonlinear, spectrally constrained inversion of the finite-element representation of the standard diffusion approximation using the Tikhonov-regulated Gauss–Newton approach to reconstruct optical properties at each node of the coarse mesh. These optical properties were used to generate the reconstructed images in this paper.

To use breast composition and the lesion shape and location for prior-guided reconstructions, each 1 mm slice of the DBT breast outline was registered to the corresponding depth of the DOT mesh contour using an affine transformation described in our prior work.^49^ A two-composition structural prior comprising the likelihood of adipose and fibroglandular tissue at each tetrahedral node was generated based on the DBT image stack using a histogram-based segmentation method described previously.^53^ The lesion markings on DBT slices were transformed to the DOT imaging space through the contour-based registration, and their outlines and 3D centroids were then extracted, from which a three-composition structural prior with an additional lesion mask was generated. When solving the inverse problem, composition structural priors were used as soft constraints in our prior-guided reconstruction algorithm described previously.^53^

To account for shifts in breast position and non-elastic deformation between the two separate compressions for optical and X-ray data acquisition, the 3D location of the lesion used for the three-composition prior-guided reconstruction was further adjusted using the three-step lesion scanning method previously described.^54^ In short, potential lesion locations were placed in grid locations in the mesh, with the scanning range shrinking with each step, to pinpoint the most plausible location by maximizing the recovery of lesion contrast.

### 2.4 Image Analysis

At each imaging session, following image reconstruction, the total hemoglobin concentration (HbT) and tissue hemoglobin oxygenation (${\mathrm{StO}}_{2}$) values were extracted from the lesion and background region for each 3-s frame of the 1-min acquisition time of each compression period. To allow for monitoring hemodynamic changes in tumor longitudinally in response to NACT, values within the lesion region at later time points, i.e., pre-cycle 2 and pre-cycle 3, were computed within the outline of the baseline lesion marking but shifted such that the centroid of the baseline marking was aligned with the centroid of the marking for that specific session (see dotted white outlines in Figs. 4 and 5). To obtain tumor and background values, the mean value of all reconstruction mesh nodes within the lesion marking and the median value of those within the remaining tissue background, respectively, were calculated, excluding nodes within 1 cm from the top or bottom surfaces of the mesh or within the extended chest wall region between −2 and 0 cm on the x-axis. Lesion values were normalized using the corresponding background in the same breast to account for confounding background variations across patients caused by physiological factors, such as breast density, menstrual cycle,^55^^,^^56^ etc., or by treatment-induced changes in normal breast tissues.^57^^,^^58^ Specifically, normalized HbT was quantified as the ratio of the mean value within the lesion divided by the median of the background, i.e., ${\overset{¯}{\mathrm{HbT}}}_{\text{tumor}}/{\overset{˜}{\mathrm{HbT}}}_{\mathrm{bg}}$; whereas normalized ${\mathrm{StO}}_{2}$ was quantified as the difference between the mean value within the lesion and the median of the background, i.e., ${\overset{¯}{\mathrm{StO}2}}_{\text{tumor}}-{\overset{˜}{\mathrm{StO}2}}_{\mathrm{bg}}$. The median of the background values was used because, compared to the mean, this summary statistic is less prone to bias toward outliers that occasionally occur on a few reconstruction nodes. As a sensitivity analysis, we also tested the use of the mean of the background for normalization purposes and we observed no change in group analysis conclusions. Finally, the median values of the normalized HbT and ${\mathrm{StO}}_{2}$ across ∼20 frames of the 1-min DOT acquisition at each compression level were used as DOT-derived tumor metrics for each period in statistical analysis.

To compare with standard clinical imaging modalities, lesion size-based metrics were also quantified. On DBT, since the breast radiologist marked representative frames of lesions at various depths in each patient case, markings were first collapsed in a two-dimensional projection, and then the cross-sectional area of the merged lesion marking was calculated. For patients with clinical MRI available at both baseline and pre-cycle 3, the length of the primary lesion in the longest dimension was used, as reported by the breast radiologist (MAS) per standard clinical practice.

### 2.5 Statistical Analysis

Summary statistics, such as the mean, median, standard deviation, and range, were computed and visualized using box plots for DOT-derived continuous metrics. Spaghetti plots were also utilized to visualize trend changes for temporal data. Based on the pathological evaluation of surgically removed tissue upon completion of NACT, the patients were grouped into two outcomes: those who achieved a pCR and those who had residual cancer (non-pCR).

Informed by prior work in the field, the primary aim of this study was to validate whether the normalized HbT under half mammographic compression, i.e., period 1, at pre-cycle 3 differs between patients who do and do not achieve pCR. To this end, the p-value of the Mann–Whitney Wilcoxon test using a two-sided hypothesis was calculated to assess differences in the distributions of HbT values by pCR status. In addition, we performed exploratory analyses to assess the predictive performance of this primary DOT-derived imaging marker. For this purpose, logistic regression was first used to model the association between the normalized HbT during period 1 at pre-cycle 3 and the post-treatment pCR status. Then, an internally validated receiver operating characteristic (ROC) curve using leave-one-out cross-validation (LOOCV) was derived and summarized using the area under the ROC curve (AUC) and the associated 95% confidence interval (CI). For comparison, the above analyses were repeated for changes in tumor area on DBT and tumor size on MRI, respectively.

The secondary aims of this study were to explore if compression-induced changes in DOT-derived metrics were predictive of pCR and if the prediction could be made at an earlier time, such as pre-cycle 2. These exploratory aims were motivated by findings from prior studies that reported tissue oxygenation ${\mathrm{StO}}_{2}$ as an early indicator of pCR^23^^–^^25^ and that hemodynamic changes during mammographic compression are correlated with pathologic outcomes.^45^ To this end, we explored other possible imaging markers which consisted of a full combination of the normalized HbT and ${\mathrm{StO}}_{2}$ values at either a single imaging time point (i.e., pre-cycle 2 and pre-cycle 3) or changes across two imaging time points (i.e., baseline versus pre-cycle 2, and baseline versus pre-cycle 3) using either absolute normalized values at each compression period (i.e., periods 1 to 3) or cross-period changes (i.e., period 1 versus period 2, and period 2 versus period 3). This resulted in a total of 40 different comparisons using the same statistical analysis described above. To account for multiple comparisons in this exploratory aim, p-values were adjusted using the Benjamini and Hochberg false discovery rate correction.

All statistical computations were done using MATLAB 2023a (MathWorks, Inc.) and RStudio v2022.12.0+353 (Posit Software).^59^ P-values smaller than 0.05 were considered statistically significant.

## 3 Results

### 3.1 Patient Enrollment Information

**Fig. 3:**
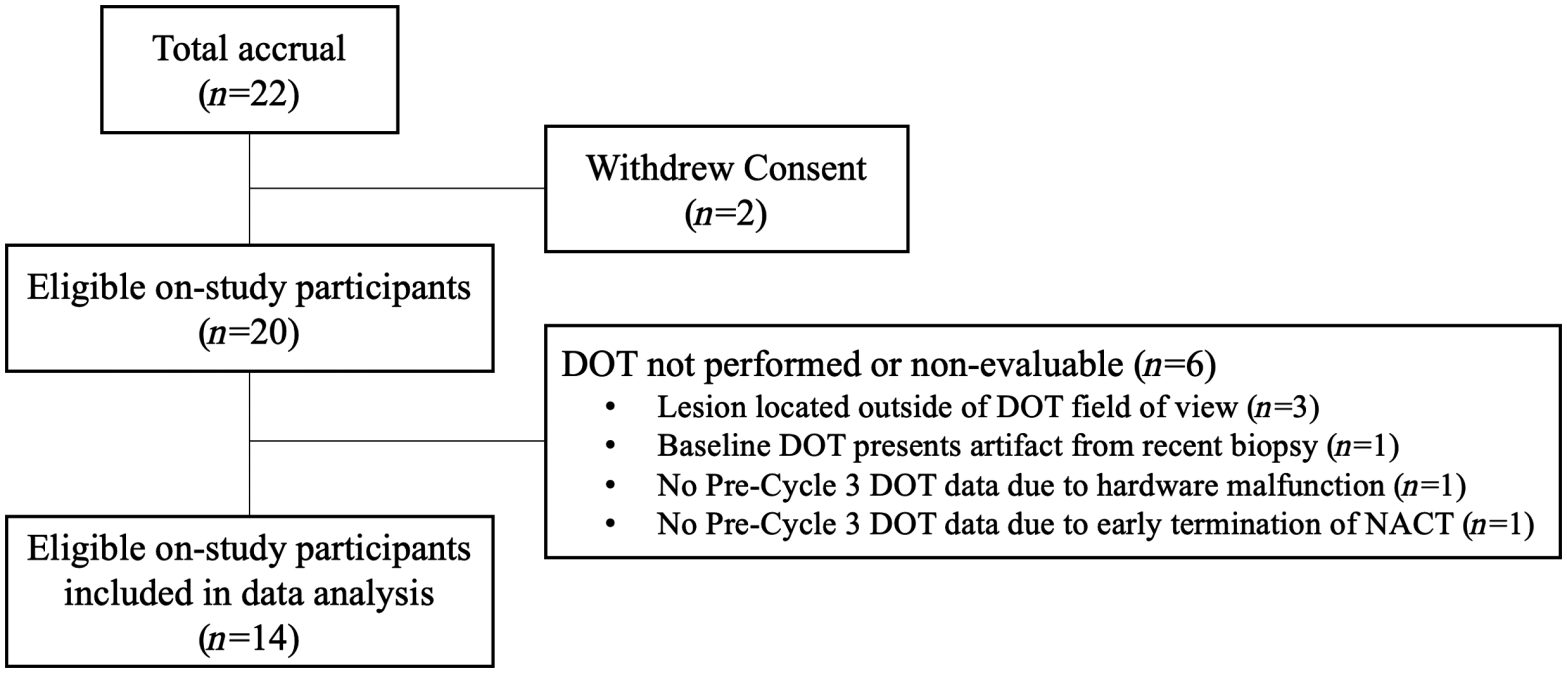
Study enrollment flowchart.

A total of 22 patients who met the eligibility criteria were enrolled. As shown in Fig. 3, two patients withdrew before initiating the imaging study. Altogether, measurements at the two required imaging sessions to assess the aims of the study were performed in 91% and 82% of all enrolled patients at baseline and pre-cycle 3, respectively. However, six patients were excluded from group data analysis due to various issues during the imaging procedures: primary tumor located outside the DOT field of view (n=3), mandatory baseline DOT presented with an artifact from recent biopsy (n=1), mandatory pre-cycle 3 DOT not performed due to imaging hardware malfunction (n=1), and early termination of the patient’s NACT regimen (n=1). Thus, the final group analysis included 14 patients with complete, evaluable DOT/DBT imaging data at both required imaging sessions. Among them, 10 patients also opted for an optional DOT/DBT imaging session at pre-cycle 2, and 8 patients had an optional clinical MRI scan at pre-cycle 3.

### 3.2 Patient, Tumor, and Treatment Characteristics

Table 1 presents the patient and tumor characteristics of all patients included in the analysis of the primary aim (n=14). Among analyzed patients, the average age was 49.7±11.5 years (range 33 to 68 years), and the breast density distribution was 29% scattered dense (BI-RADS B) and 71% heterogeneously dense (BI-RADS C). The racial distribution was 86% White, 7% Black, and 7% Asian; 93% were of non-Hispanic or -Latina ethnicity. Baseline tumor characteristics were also recorded. All patients included in the final analysis were diagnosed with a primary lesion of invasive ductal carcinoma type, and 86% of them were TN type.

**Table 1:** Characteristics of patients, primary tumor, and treatment outcome of patients included in group analysis.

ID	Age	Race	Ethnicity	BI-RADS breast density	Primary lesion size on MRI at diagnosis (mm)	Receptor status	Treatment regimen	Treatment outcome	Opted in Pre-C2 DOT?	Opted in Pre-C3 MRI?
1	51	White	Non-Hispanic	C	92 × 67 × 78	ER-/PR-/HER2-	AC-T	RCB-I	Yes	No
2	42	White	Non-Hispanic	C	21 × 18 × 20	ER-/PR-/HER2-	AC-T	RCB-II	Yes	No
3	67	White	Non-Hispanic	B	31 × 25 × 23	ER-/PR-/HER2-	PC	pCR	Yes	No
4	33	White	Non-Hispanic	C	45 × 26 × 26	ER-/PR-/HER2+	THP	pCR	Yes	No
5	62	White	Non-Hispanic	C	27 × 19 × 24	ER-/PR-/HER2-	AC-T	RCB-II	Yes	No
6	42	White	Non-Hispanic	C	25 × 21 × 34	ER-/PR-/HER2-	PC + Pembrolizumab	RCB-II	No	Yes
7	42	White	Non-Hispanic	C	30 × 32 × 32	ER-/PR-/HER2-	NeoSTAR	RCB-I	Yes	Yes
8	68	White	Non-Hispanic	B	19 × 13 × 12	ER-/PR-/HER2-	NeoSTAR	RCB-II	No	Yes
9	45	White	Non-Hispanic	C	13 × 10 × 10	ER-/PR-/HER2-	NeoSTAR	pCR	Yes	Yes
10	46	African American	unknown	C	16 × 14 × 17	ER-/PR-/HER2+	THP	pCR	No	Yes
11	45	White	Non-Hispanic	B	23 × 18 × 14	ER-/PR-/HER2-	NeoSTAR	pCR	Yes	Yes
12	47	White	Non-Hispanic	C	25 × 20 × 17	ER-/PR-/HER2-	NeoSTAR	RCB-II	Yes	Yes
13	67	Asian	Non-Hispanic	C	34 × 31 × 42	ER-/PR-/HER2-	TAC	RCB-II	No	Yes
14	39	White	Non-Hispanic	B	58 × 46 × 42	ER-/PR-/HER2-	P-RAD	RCB-I	Yes	No

ER, estrogen receptor; PR, progesterone receptor; HER2, human epidermal growth factor receptor 2; AC-T, adriamycin (doxorubicin) + cytoxan (cyclophosphamide) followed by Taxol (paclitaxel); PC, paclitaxel (Taxol) + carboplatin; THP, Taxol (paclitaxel) + herceptin (trastuzumab) + Perjeta (pertuzumab); NeoSTAR (clinical trial), sacituzumab govitecan (patient #7 also followed by PC); TAC, taxotere (docetaxel) + adriamycin (doxorubicin) + cytoxan (cyclophosphamide); P-RAD (clinical trial), PC + pembrolizumab followed by AC followed by radiation boost; RCB, residual cancer burden.

Patients were recruited regardless of the NACT treatment regimen received. Although the two HER2+ patients received the same THP regimen with targeted agents, the treatment protocols for the 12 TN patients were heterogeneous, including five who participated in the NeoSTAR trial and received sacituzumab govitecan.^60^ Nevertheless, treatment regimens for all patients were based on cytotoxic therapies, and each cycle was 3 weeks long. Upon completion of the NACT, the pathological response was evaluated on surgically removed tissue. Five patients achieved pCR (36%) or residual cancer burden category 0 (RCB-0), 3 achieved RCB-I (21%), and 6 achieved RCB-II (43%) as the final pathological outcome. RCB-I and RCB-II cases were further grouped into one non-pCR category (63%) for statistical analysis. There were no significant differences in age, breast density, and lesion size at diagnosis between the pCR and non-pCR groups.

While subject to compression during DOT imaging, the mean ± standard deviation and the range of breast thickness of all 14 patients were 9.0±0.7  cm (range 7.8 to 9.9 cm), 8.6±0.7  cm (range 7.5 to 9.6 cm), and 8.7±0.7  cm (range 7.5 to 9.6 cm) for the half, full, and half compression periods (i.e., periods 1 to 3), respectively, at baseline. At pre-cycle 3, those values were 8.3±0.7  cm (range 7.1 to 9.5 cm), 7.9±0.7  cm (range 6.7 to 9.2 cm), and 8.0±0.7  cm (range 6.7 to 9.2 cm) for periods 1–3, respectively. The forces used to maintain the above breast thickness were 8.1±2.4  lbf (range 4.5 to 13.4 lbf), 16.7±5.5  lbf (range 7.5 to 29.8 lbf), and 8.3±2.4  lbf (range 4.6 to 13.5 lbf) for periods 1 to 3, respectively, at baseline. At pre-cycle 3, those values were 8.4±1.9  lbf (range 5.3 to 11.7 lbf), 17.9±4.0  lbf (range 10.6 to 27.6 lbf), and 9.9±3.3  lbf (range 5.0 to 16.6 lbf) for periods 1 to 3, respectively. There was no significant difference in force between baseline and pre-cycle 3 at any of the compression period. However, the breast thickness was significantly different at all three compression periods between baseline and pre-cycle 3. This may be attributed to treatment-induced changes in gross properties of breast tissue as reported by others^57^ or weight loss. In this study, all DOT-derived biomarkers were normalized to minimize the impact of such systematic changes.

### 3.3 Longitudinal DOT/DBT Imaging

**Fig. 4:**
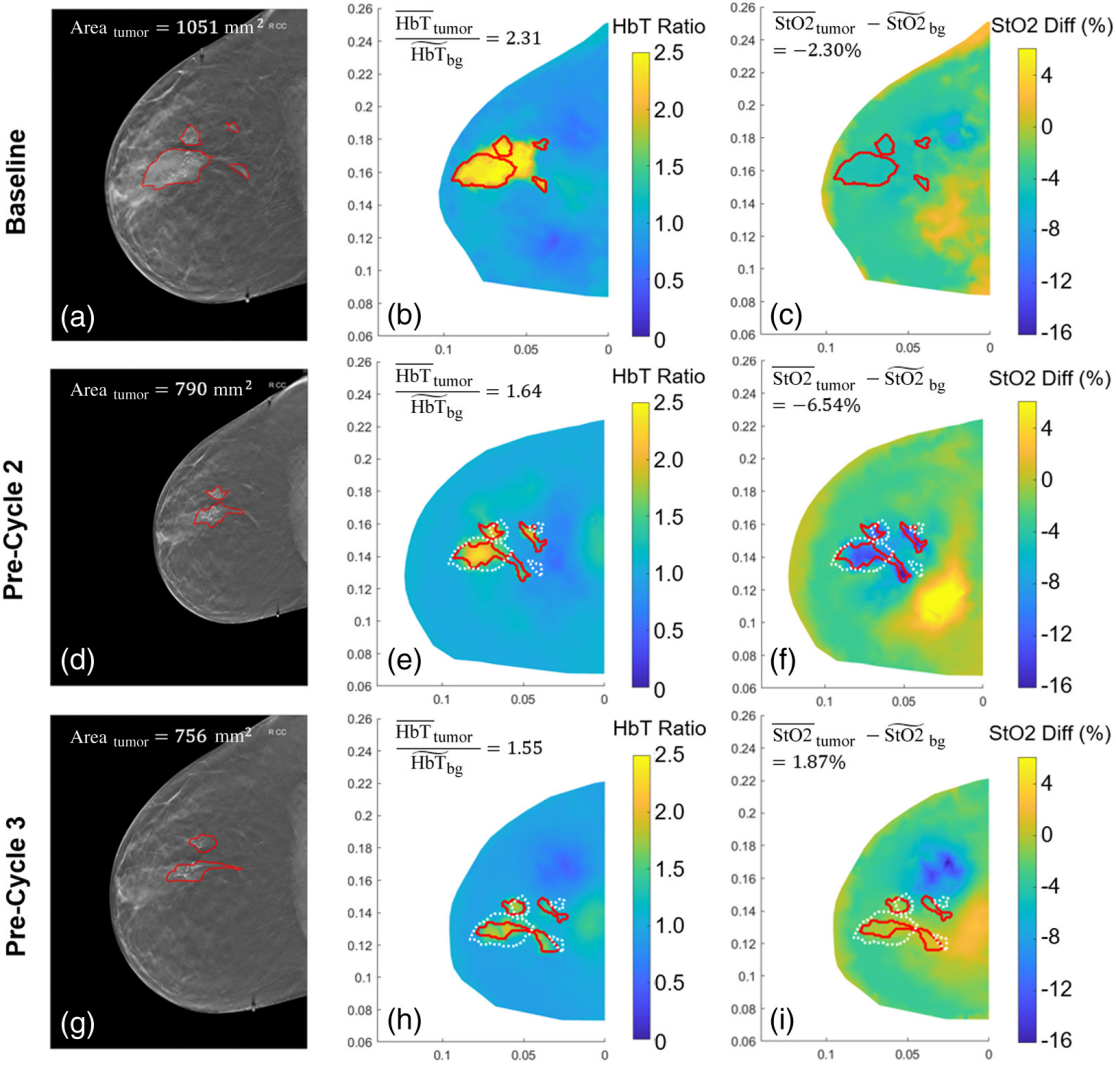
Longitudinal image panel of a pCR case at three time points along the course of NACT. (a), (d), (g) DBT with radiologic tumor marking by breast radiologist (MAS) in red; (b), (e), (h) normalized HbT and (c), (f), (i) normalized ${\mathrm{StO}}_{2}$ maps at half mammographic compression during period 1. Red solid line on HbT and ${\mathrm{StO}}_{2}$ images: tumor marking of each imaging session transformed from that on DBT; White dotted line: transformed baseline tumor marking used to calculate the normalized HbT and ${\mathrm{StO}}_{2}$ within the tumor as noted in the upper left corner of each panel.

**Fig. 5:**
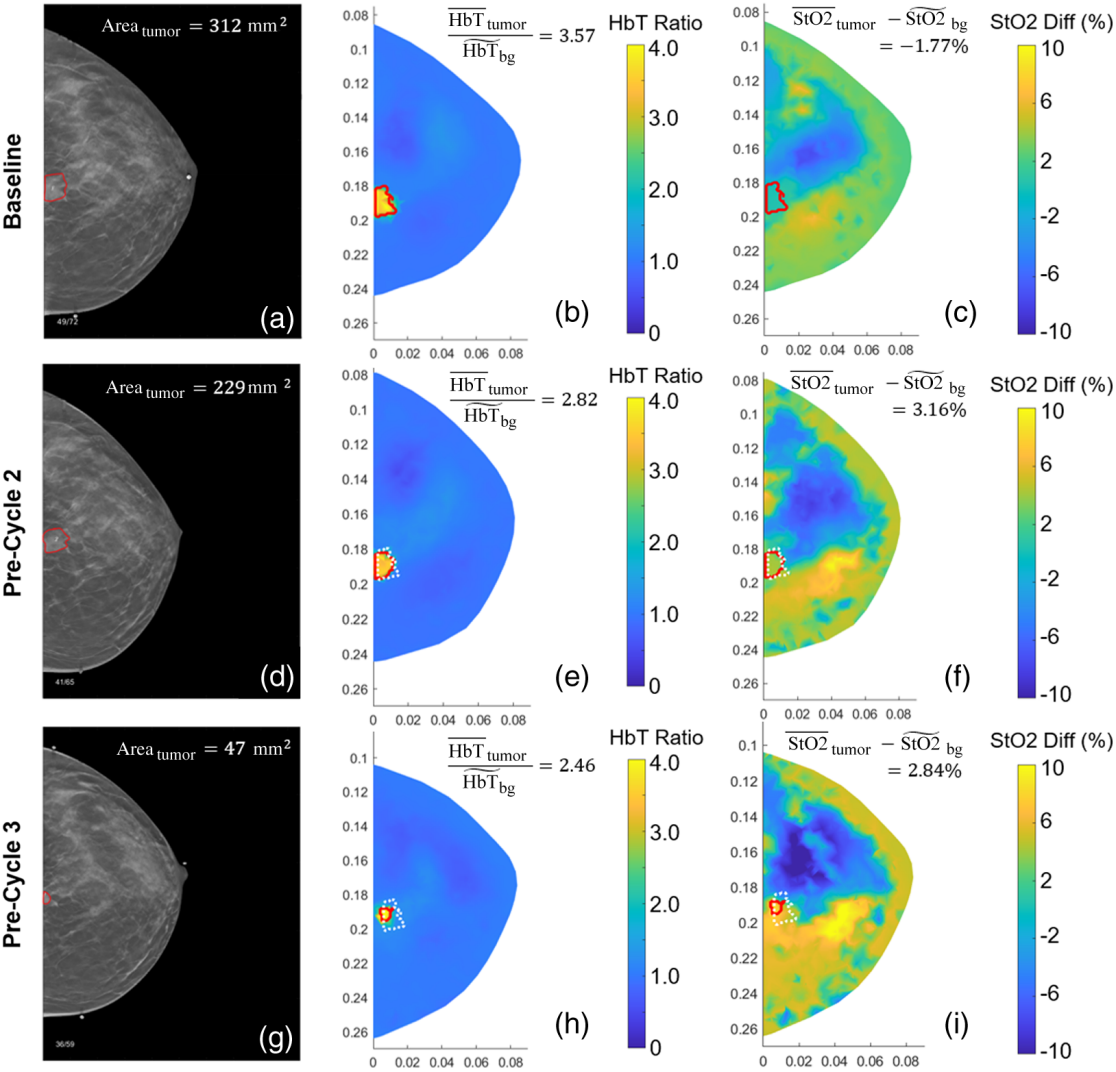
Longitudinal image panel of a non-pCR case at three time points along the course of NACT arranged the same way as in Fig. 4.

Example multimodal DOT/DBT images at three time points (baseline, pre-cycle 2, and pre-cycle 3) along the course of NACT of two patients, one pCR and the other non-pCR, are shown in Figs. 4 and 5, respectively. As shown in Figs. 4(b) and 5(b), at baseline, both patients exhibited high HbT with over twice the concentration in the tumor compared to the background. In response to NACT, as expected, the tumor HbT level in both cases started to drop, accompanied by progressive shrinkages in tumor size. At pre-cycle 2, the normalized HbT reduced to 1.64 and 2.82 from the baseline levels of 2.31 and 3.57 in the pCR and non-pCR cases, respectively, and the total tumor area quantified on DBT reduced by 25% and 27% accordingly. However, as shown in Fig. 4(h), at pre-cycle 3, the HbT level within the tumor region is no longer significantly higher than the surrounding tissue in the pCR case, whereas in the non-pCR case shown in Fig. 5(h), though tumor size shrank substantially by 85%, the normalized HbT remained at a high level of 2.46.

When assessing tissue oxygenation, as shown in Figs. 4(c) and 5(c), at baseline, the tumor in both cases showed negative normalized ${\mathrm{StO}}_{2}$ values, i.e., −2.30% in the pCR case in Fig. 4(c) and −1.77% in the non-pCR case in Fig. 5(c), which suggested lower tissue oxygenation levels within the tumors compared to the background. However, as early as pre-cycle 2, tumor ${\mathrm{StO}}_{2}$ for the non-pCR case started increasing and maintained at a higher level at pre-cycle 3, resulting in positive normalized tumor ${\mathrm{StO}}_{2}$ values of 3.16% and 2.84% at the two time points, respectively, as shown in Figs. 5(f) and 5(i). In comparison, the pCR case sustained a negative normalized tumor ${\mathrm{StO}}_{2}$ of −6.54% at pre-cycle 2 before reaching a positive value of 1.87% at pre-cycle 3, as shown in Figs. 4(f) and 4(i).

### 3.4 Primary Marker: Normalized HbT at Half Mammographic Compression

**Fig. 6:**
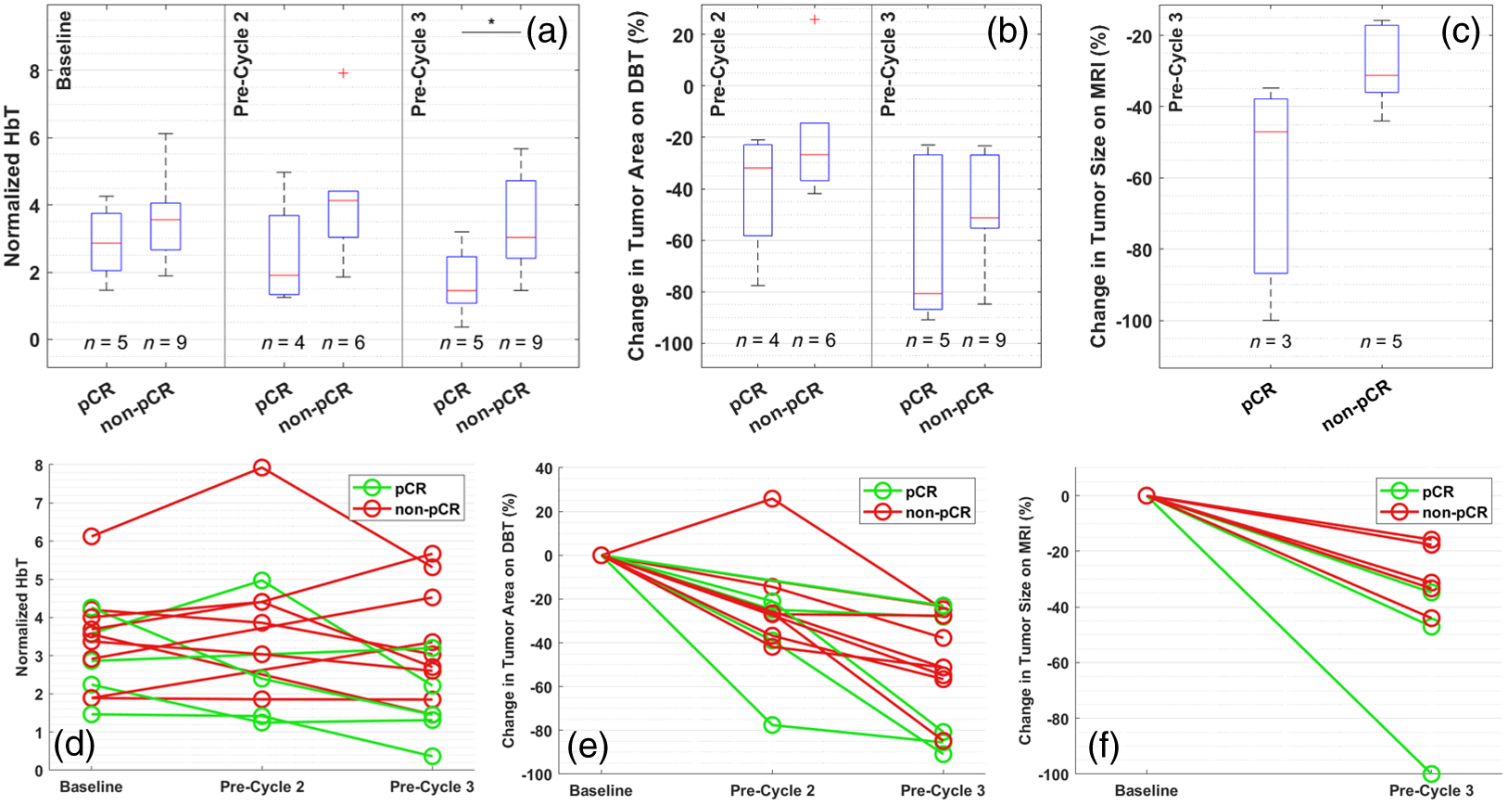
Box and line plots of (a), (d) normalized HbT at half mammographic compression during period 1, (b), (e) percentage change of tumor area on DBT, and (c), (f) percentage change of tumor size on MRI at longitudinal time points. The bottom and top edges of each box indicate the 25th and 75th percentiles, respectively, and the red line in each box indicates the median. Significance bar of two-sided two-sample Mann–Whitey U-test between pCR and non-pCR: *p<0.05 (unadjusted).

Figures 6(a) and 6(d) show the differences between pCR and non-pCR groups, depicted in both box and spaghetti plots, using the primary DOT-derived imaging marker, i.e., normalized HbT at half mammographic compression during period 1 (see measurement protocol in Fig. 2). In general, the pCR cases demonstrated steady decreases in normalized tumor HbT to 1.91 [IQR: 1.33 to 3.68] (median [IQR: interquartile range]) and 1.45 [IQR: 1.08 to 2.46], respectively, at pre-cycle 2 and pre-cycle 3, which is 76.56% [IQR: 55.98 to 117.84%] and 58.58% [IQR: 31.78 to 74.34%] of the baseline level. In contrast, the non-pCR cases showed various degrees of progressive or stable normalized tumor HbT levels over time to 4.13 [IQR: 3.04 to 4.41] and 3.04 [IQR: 2.41 to 4.72], respectively, at pre-cycle 2 and pre-cycle 3, which is 104.12% [IQR: 91.87 to 119.50%] and 86.80% [IQR: 71.05 to 154.03%] of the baseline level. At pre-cycle 3, the normalized HbT was significantly different between the two groups (p=0.042).

On the other hand, as shown in Figs. 6(b)–6(c) and 6(e)–6(f), the tumor size defined on either DBT or clinical MRI visibly decreased in all cases. Compared to non-pCR, pCR cases also saw a higher percentage of tumor shrinkage, especially at pre-cycle 3 (−80.78% [IQR: −86.89 to −26.79%] for pCR versus −51.27% [IQR: −55.31 to −26.88%] for non-pCR on DBT and −47.06% [IQR: −86.76 to −37.85%] for pCR versus −31.25% [IQR: −36.00 to −17.18%] for non-pCR on MRI). However, we did not detect significant differences between the two outcome groups at any follow-up imaging time points using size-based metrics.

### 3.5 Compression-Induced Changes in Normalized ${\mathrm{StO}}_{2}$

**Fig. 7:**
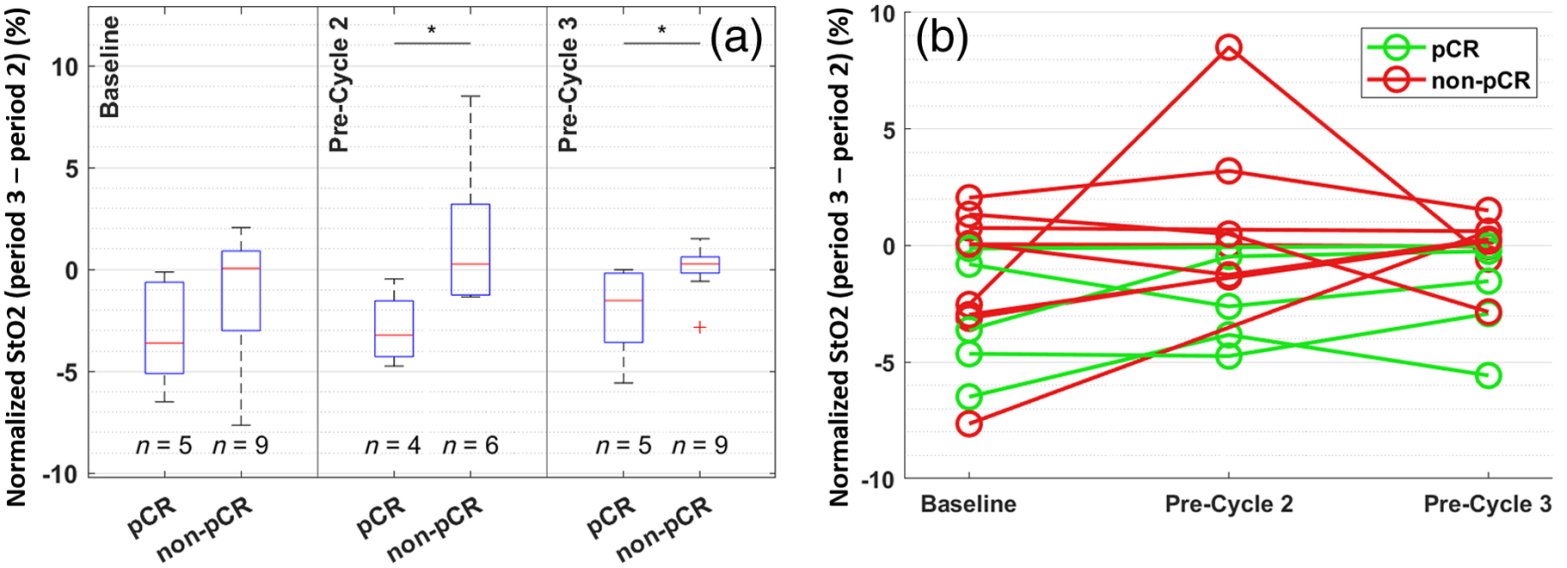
Box (a) and line (b) plots of period 3 versus period 2 difference in normalized ${\mathrm{StO}}_{2}$ at longitudinal time points. The bottom and top edges of each box indicate the 25th and 75th percentiles, respectively, and the red line in each box indicates the median. Significance bar shows the result of a two-sided two-sample Mann–Whitey U-test between pCR and non-pCR: *p<0.05 (unadjusted).

Beyond the primary DOT-derived response marker shown above, using our exploratory statistical analysis detailed in Sec. 2.5, the change in normalized tissue oxygenation upon releasing the force from full mammographic compression in period 2 to half in period 3 was identified as another potentially effective imaging marker. At baseline, upon reducing pressure by half from period 2 to period 3, there was a tendency toward decrease in the normalized tumor ${\mathrm{StO}}_{2}$ in most cases, resulting in negative values as shown in Fig. 7(b). However, the median of change from period 2 to period 3 in normalized tumor ${\mathrm{StO}}_{2}$ was negative (−3.61%) in the pCR group whereas that in the non-pCR group was close to zero (0.06%). In the pCR group, the magnitude of such compression-induced changes in normalized ${\mathrm{StO}}_{2}$ reduced over time, as evidenced by the median values gradually approaching 0 from −3.61% at the baseline to −1.52% at pre-cycle 3 in Fig. 7(a). In comparison, the reduction in compression-induced changes in ${\mathrm{StO}}_{2}$ returned to 0 much faster in the non-pCR group with some even overshooting into positive changes at pre-cycle 2. At pre-cycle 3, the reduction in compression force from period 2 to period 3 resulted in little change (0.27% [IQR: −0.17 to 0.61%]) in normalized ${\mathrm{StO}}_{2}$ in the non-pCR group, compared to the persistent negative change observed in the pCR group (−1.52% [IQR: −3.58 to −0.18%]). Such distinctions between the two outcome groups are significant at both pre-cycle 2 (p=0.038) and pre-cycle 3 (p=0.029).

### 3.6 Association and Predictive Ability of Pathological Outcome

**Fig. 8:**
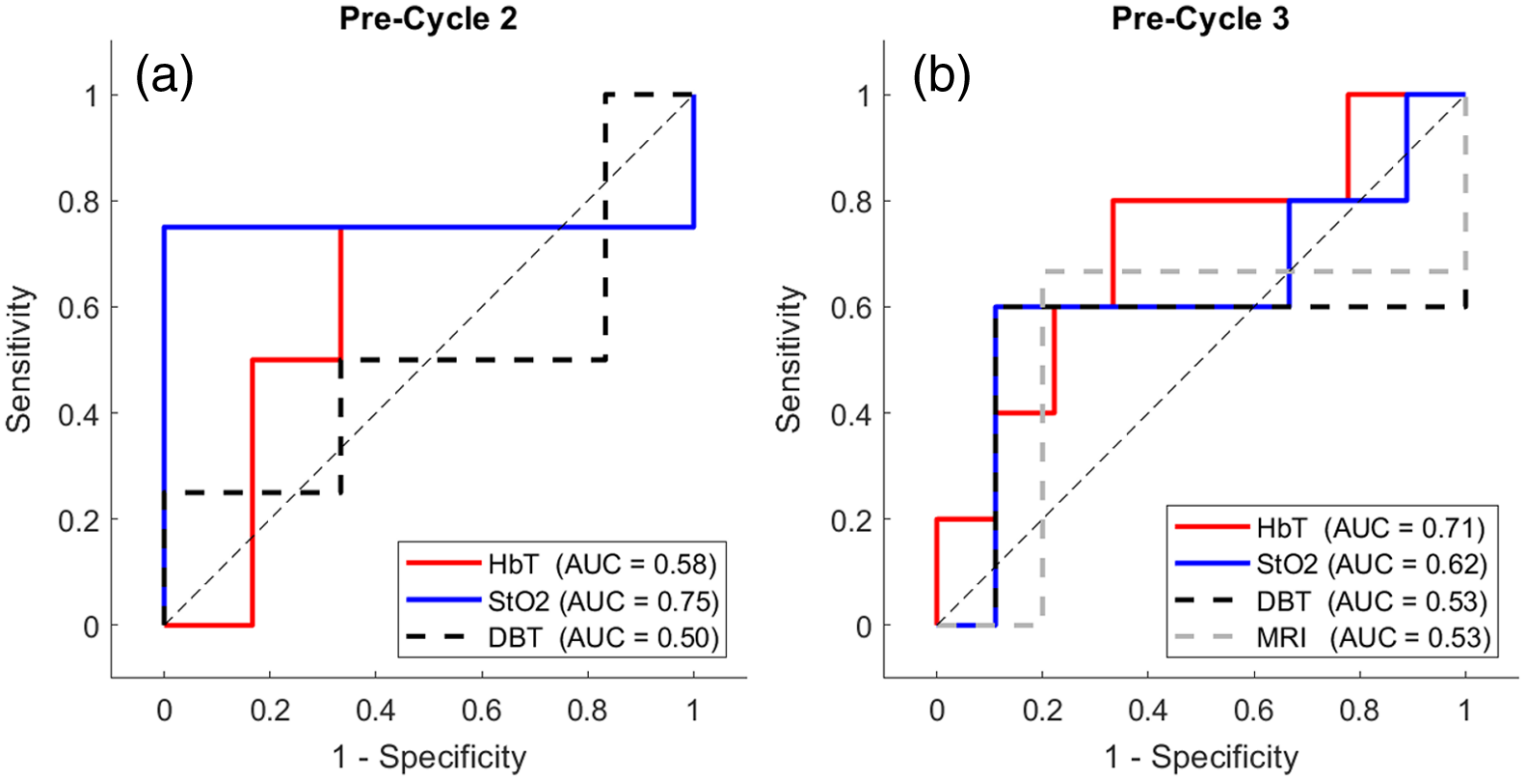
ROC curves of DOT-derived and size-based imaging markers at (a) pre-cycle 2 and (b) pre-cycle 3, respectively. AUC values are noted in the legend.

Tables 2 and 3 show p-values of comparisons between pCR and non-pCR groups, as well as the AUCs and the corresponding CIs using various DOT-derived and DBT/MRI size-based imaging markers. Figure 8 plots the ROCs at pre-cycle 2 and pre-cycle 3, respectively, for comparison of performance at each time point. At pre-cycle 3, normalized HbT at half compression reached an estimated AUC of 0.71 (95% CI: [0.40 to 1.00]), comparing to the AUC estimates of 0.53 (95% CI: [0.09 to 0.98]) and 0.53 (95% CI: [0.00 to 1.00]), respectively, using DBT- and MRI-based tumor size markers. At pre-cycle 2, after adjusting for multiple comparisons, the differences between pCR and non-pCR groups were no longer significant using the secondary ${\mathrm{StO}}_{2}$ marker, i.e., the change in normalized tissue oxygenation upon releasing the force from full mammographic compression in period 2 to half in period 3. However, the estimated AUC using the ${\mathrm{StO}}_{2}$ marker remained as high as 0.75 (95% CI: [0.26 to 1.00]) at pre-cycle 2, comparing to only 0.50 (95% CI: [0.04 to 0.96]) using the percentage change in tumor area measured on DBT. Notably, as shown in Table 3, the AUC of the compression-induced differences in normalized ${\mathrm{StO}}_{2}$ is estimated higher at pre-cycle 2 than at pre-cycle 3 (0.62, 95% CI: [0.25 to 1.00], indicating its potential as an early predictor of NACT.

**Table 2:** Comparison of performance statistics between the primary DOT-derived functional imaging marker, i.e., normalized HbT at half compression, and lesion size-based morphological imaging markers. IQR, interquartile range; CI, confidence interval.

Metrics	Time point	Median [IQR]		p-value	Internally validated AUC estimate [95% CI]
		pCR	Non-pCR		
Normalized HbT @ period 1	Pre-cycle 3	1.45 [1.08 to 2.46]	3.04 [2.41 to 4.72]	0.042	0.71 [0.40 to 1.00]
% change in tumor area on DBT	Pre-cycle 3	−80.78% [−86.89 to −26.79%]	−51.27% [−55.31 to −26.88%]	0.438	0.53 [0.09 to 0.98]
% change in tumor size on MRI	Pre-cycle 3	−47.06% [−86.76 to −37.85%]	−31.25% [−36.00 to −17.18%]	0.071	0.53 [0.00 to 1.00]

**Table 3:** Performance statistics of exploratory imaging markers. IQR, interquartile range; CI, confidence interval.

Metrics	Time point	Median (IQR)		Adjusted p-value	Internally validated AUC estimate [95% CI]
		pCR	Non-pCR		
Normalized HbT @ period 1	Pre-cycle 2	1.91 [1.33 to 3.68]	4.13 [3.04 to 4.41]	0.570	0.58 [0.13 to 1.00]
Normalized ${\mathrm{StO}}_{2}$ (period 3 to period 2)	Pre-cycle 2	−3.22% [−4.28 to −1.54%]	0.26% [−1.25 to 3.21%]	0.509	0.75 [0.26 to 1.00]
	Pre-cycle 3	−1.52% [−3.58 to −0.18%]	0.27% [−0.17 to −0.61%]	0.509	0.62 [0.25 to 1.00]
% change in tumor area on DBT	Pre-cycle 2	−31.92% [−58.29 to −22.92%]	−26.72% [−36.77 to −14.41%]	0.777	0.50 [0.04 to 0.96]

## 4 Discussion

Breast DOT is a functional imaging modality that can provide hemoglobin-based imaging markers to monitor tumor response to NACT. Building on prior work, this study investigates if early predictions of pCR can be made using multimodal DOT/DBT instrumentation, which would facilitate integration of DOT into clinical practice given that DBT is widely used for breast imaging. Since breasts are subject to mammography-like compression, we further investigated if variations in the compression level can provide additional information on the tumor response to therapy. Compared to standalone breast DOT that was often used previously for the same purpose, this multimodal imaging approach allows us to utilize clinical 3D DBT, contemporaneously acquired with the same patient positioning protocol as in CC-view X-ray mammography, as structural priors to enhance the contrast recovery of breast lesions. By implementing a semi-automated three-step lesion scanning image registration method developed by us previously,^54^ we ensured accurate tumor localization and consistent DOT images across time points for longitudinal data analysis. Moreover, our imaging protocol (shown in Fig. 2) involved compressing the breast at two pressure levels in three 1-min DOT acquisition periods. This enabled us to explore the role of compression-induced hemodynamic DOT markers, previously demonstrated useful in the differential diagnosis of breast cancer, in monitoring therapy outcomes.^35^^–^^44^ Also, at each imaging session, the study breast radiologist marked the tumor outlines on DBT images acquired immediately after the DOT imaging, providing a standard-of-care NACT response evaluation metric based on changes in anatomic tumor size as the reference for comparison with DOT-derived imaging markers. Another distinction of our study from many prior ones is the use of more stringent AUC calculations based on LOOCV for a more realistic estimation of the predictive power of DOT imaging markers despite a relatively small sample size.

Based on prior work in the field, normalized HbT at pre-cycle 3, i.e., the mid-treatment time point, was analyzed as the primary DOT-derived imaging marker in this clinical study. In particular, since compression is expected to reduce the blood content within the breast, we have focused on the normalized HbT in period 1, where breasts were compressed at half mammographic compression. As expected, normalized HbT during period 1 at pre-cycle 3 was indeed significantly different (p=0.042) between pCR and non-pCR groups despite the relatively small sample size of 14 as shown in Fig. 6(a). At baseline, doubling the force to full mammographic compression in period 2 resulted in minimal changes in the absolute HbT values in background (−0.43±5.42%, p=0.93) and a moderate but significant decrease in the normalized tumor HbT (−13.92±17.76%, p=0.02) in all patients. Upon releasing back to half compression from period 2 to period 3, the breasts stayed at about the same thickness as they were under full compression, and there were insignificant increases in tumor (5.98±3.52%, p=0.19) and background (1.52±3.52%, p=0.13) HbT. However, at pre-cycle 3, when most cases saw a decrease of tumor HbT at various levels in response to NACT, doubling the compression no longer led to a significant reduction in normalized HbT (−4.47±14.31%, p=0.42). While exploring other potential imaging markers, we observed no significant differences between pCR and non-pCR groups using normalized tumor HbT values in period 2 or period 3 either by themselves or by taking cross-period differences as compression-induced response markers.

On the other hand, compared to HbT-based metrics, tissue oxygenation-based metrics, i.e., normalized ${\mathrm{StO}}_{2}$, did not exhibit strong associations with pCR by themselves. However, we found that compression-induced changes in ${\mathrm{StO}}_{2}$ from period 2 to period 3, i.e., upon reducing the compression force from full to half, were associated with eventual pCR status. The observed difference could be caused by changes in tumor vasculature or the formation of scar tissue in response to NACT that lead to disproportional re-oxygenation upon partial decompression. In particular, at pre-cycle 2, the discriminatory ability of the period 3 versus period 2 difference in normalized tumor ${\mathrm{StO}}_{2}$ (AUC=0.75) is comparable to that of normalized tumor HbT in period 1 at pre-cycle 3 (AUC=0.71), demonstrating its potential as an early predictor of pCR. Prior studies have also shown that when looking at the two hemoglobin species, i.e., oxy- and deoxy-hemoglobin, separately, the prediction of pathologic outcomes can be frequently made soon after the initiation of NACT within 1 to 2 weeks.^21^^,^^61^^–^^63^ Another recent work reported prediction of pCR 10 days after initiation of NACT using normalized ${\mathrm{StO}}_{2}$.^23^ In comparison, HbT-based markers usually become predictive at a later time point, typically after the completion of the first cycle (∼3 weeks) or mid-way through the NACT course.^30^^,^^33^^,^^64^ Although prior studies are heterogeneous in study design, these findings indicate that changes in tissue metabolism and oxygenation level may precede the reduction in blood volume or tissue perfusion in response to NACT, potentially offering better prediction performance at earlier time points.

Our prior therapy monitoring study using standalone DOT has observed that hemodynamic changes driven by pressure relaxation while breasts were held in partial mammography-like compression were associated with NACT outcomes.^45^ However, after studying the time courses of dynamic DOT data in this study during both period 1 and period 3, there was insufficient evidence to conclude an association with pCR. It is possible that patient posture (standing in this study versus sitting in our previous work) and the duration of constant pressure (1 min in this study versus 2 min previously) impacted the ability of these dynamic changes to offer outcome prediction. We also note that our previous study grouped patients into responders and non-responders defined by a 50% reduction in tumor size, rather than the much more stringent pCR versus non-pCR grouping used in this study. When re-analyzing the data collected in our prior study by pCR status, hemodynamic markers derived from time courses were not significantly different between pCR and non-pCR groups.

In general, as shown in Fig. 8, DOT-derived functional imaging markers outperform tumor size-based evaluation typically used in clinical practice at both pre-cycle 2 and pre-cycle 3. Changes in tumor size, either assessed on breast MRI or DBT, consistently yielded an AUC slightly above 0.5, i.e., the predictive performance is somewhat equivalent to a random guess. This is likely because all tumors imaged in this study had demonstrated various levels of shrinkage in response to treatment, as shown in Figs. 6(e) and 6(f). Since the degree of reduction in tumor size is poorly associated with pathologic outcomes, even when mid-treatment breast MRI is offered to patients undergoing NACT, treatment changes are only considered when there is no reduction in tumor size. In contrast, as shown in Fig. 8(a), DOT-derived imaging markers can potentially achieve a specificity of 1 as early as pre-cycle 2, i.e., about 3 weeks into NACT, to help achieve a personally tailored treatment regimen.

Our study has several limitations. First, all breasts were compressed in the CC view, which resulted in difficulties in positioning some tumors within the optical imaging field of view. For this reason, data from three patients were excluded from this study, which constituted 50% of all excluded cases. The decision to compress only in the CC view but not in oblique ones was made before the start of the longitudinal imaging study, as it had been observed that breast repositioning is more reliable and repeatable in the CC view. However, the findings of this study can help optimize and shorten the compression DOT imaging protocol so that other mammographic views can be used in the future to make it accessible to more patient cases. Second, compared to some prior studies,^30^^,^^32^ such as the multi-center ACRIN 6691 study,^24^ our longitudinal monitoring time points were sparse, with pre-cycle 2 as the first possible evaluation time point and pre-cycle 3 as the only required time point after the initiation of NACT. Such study design precluded us from accessing the association between DOT-derived imaging markers and pCR at very early time points, such as after the first week of NACT, as reported by others. This was limited by logistical constraints since our multimodal imaging protocol involved the use of clinical space and the DBT device. Studies using standalone DOT systems have an advantage from this perspective. Regardless, we corroborated that normalized changes in tissue ${\mathrm{StO}}_{2}$ are likely to be an earlier indicator of pCR than HbT, as reported by others, and discovered a novel compression-induced outcome marker. Finally, due to the small sample size, we could not explore multivariate models, such as combining DOT-derived imaging markers with tumor size-based ones, to further enhance the predictive performance as achieved by others.^32^ At a sample size of 14, all our statistical models were univariate. Also, since not all patients opted into the optional pre-cycle 2 scan, the analyses at pre-cycle 2 were conducted on a subset of patients who completed the pre-cycle 3 scans (N=10 versus N=14 for pre-cycle 2 versus pre-cycle 3 cohort). The difference in patient population might have contributed to differences in predicting pCR using a specific imaging marker at the two time points. Therefore, the discriminatory ability of the functional DOT-derived imaging markers discovered in this study used either by themselves or in combination with clinical factors needs to be evaluated in a large population study in the future.

## 5 Conclusion

Longitudinal multimodal DOT and DBT imaging were performed under mammographic compression on 14 breast cancer patients undergoing NACT to determine the association of hemoglobin-based functional imaging markers and pCR. Our results demonstrated that normalized tumor HbT under half compression was significantly different between pCR and non-pCR groups at pre-cycle 3. In addition, the change in normalized tumor ${\mathrm{StO}}_{2}$ upon reducing force from full to half compression was identified as another potential indicator of pCR at an earlier time point of pre-cycle 2. The predictive performance of regression models built on DOT-derived functional imaging markers outperformed those built on changes in tumor size assessed by breast MRI and DBT at both pre-cycle 2 and pre-cycle 3. These findings suggest that breast DOT can be used to assist the response assessment in women undergoing NACT, currently challenging in clinics, to potentially enable personalized adjustments of treatment regimens.

## Supplementary Material



## Data Availability

The data that support the findings of this article are not publicly available due to data sharing not being allowed per signed patient consents. They can be requested from the author at stefan.carp@mgh.harvard.edu.
