# Supplementary material for: Functional hemodynamic imaging markers for the prediction of pathological outcomes in breast cancer patients treated with neoadjuvant chemotherapy
Source: J Biomed Opt. 2024 May 11;29(6):066001. doi: 10.1117/1.JBO.29.6.066001 (PMC11088438; doi:10.1117/1.JBO.29.6.066001)
Supplement: Supplementary file 1 [file JBO_029_066001_SD001.pdf]

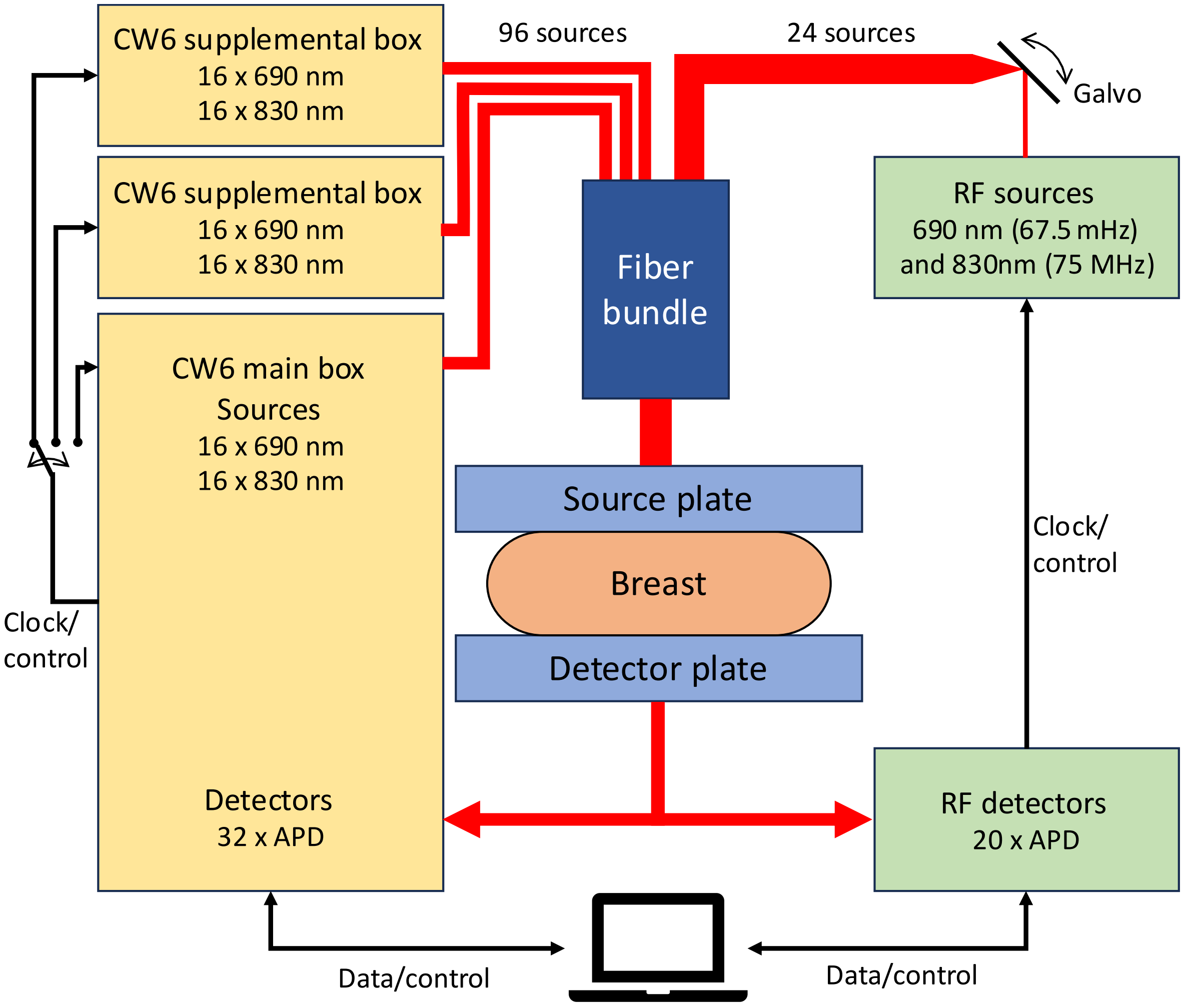

Fig. S1 (a) Schematic showing an overview of the TOBI2 breast imaging system. Near-infrared light is supplied to source locations in the compression plate by three CW source boxes and one RF source box. Transmitted light is collected by fibers on the detector plate leading to CW and RF detector boxes. Optical data acquisition is controlled by a laptop, which also stores the acquired data.

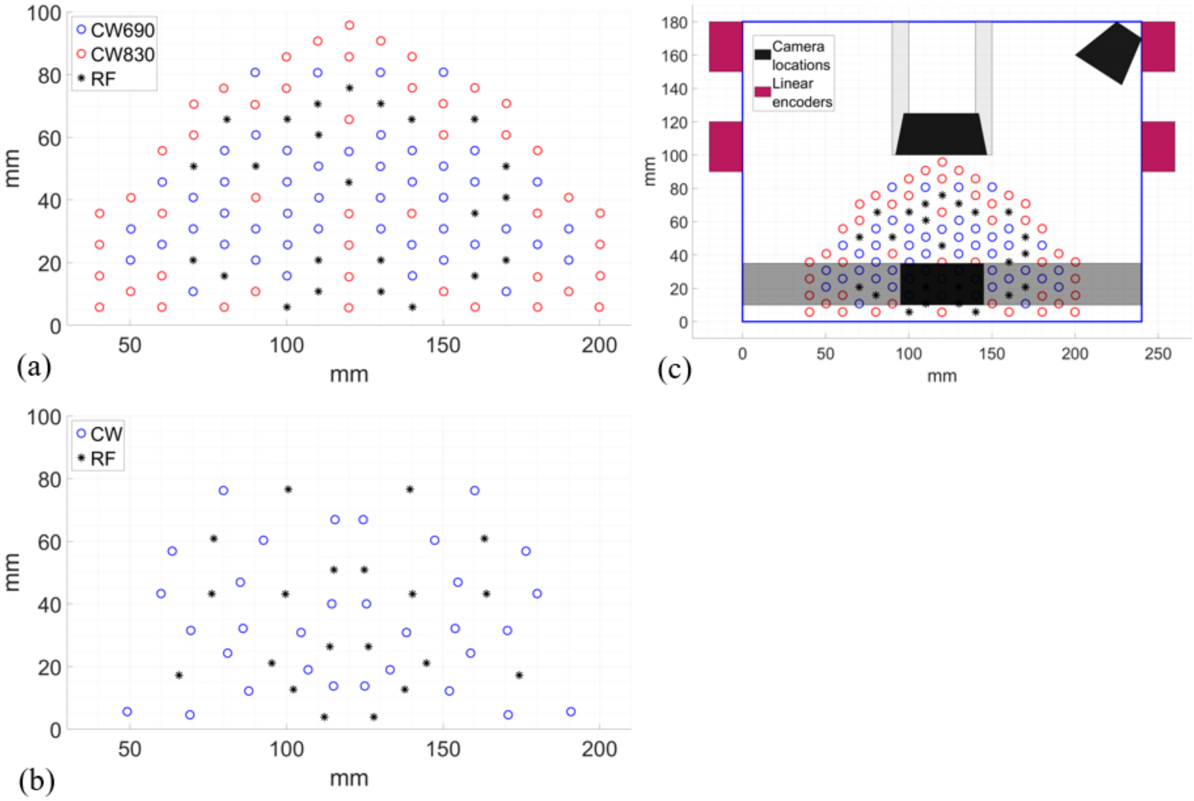

Fig. S2 (a) Layout of optical source locations on the compression plate. (b) Layout of optical detector fibers on the detector plate. (c) Full layout of compression plate with optical sources, camera locations (black) and linear encoders (magenta). Blue border marks the outer edge of the compression plate, which corresponds to the DBT field of view.
